# Human Papillomavirus (HPV) DNA Detection Using Self-Sampling Devices in Women Undergoing Long Term Immunosuppressive Therapy

**DOI:** 10.3390/v12090962

**Published:** 2020-08-30

**Authors:** Aleksandra Wielgos, Bronislawa Pietrzak, Mariusz Sikora, Gajane Martirosian, Barbara Suchonska, Jolanta Gozdowska, Urszula Oldakowska-Jedynak, Zoulikha Jabiry-Zieniewicz, Magdalena Durlik, Lidia Rudnicka, Miroslaw Wielgos

**Affiliations:** 1 Department of Dermatology, Medical University of Warsaw, 02-008 Warsaw, Poland; drmariuszsikora@gmail.com (M.S.); lidiarudnicka@gmail.com (L.R.); 2 1st Department of Obstetrics and Gynecology, Medical University of Warsaw, 02-115 Warsaw, Poland; bpietrzak@wum.edu.pl (B.P.); barbara.suchonska@wp.pl (B.S.); zzieniewicz@gmail.com (Z.J.-Z.); miroslaw.wielgos@wum.edu.pl (M.W.); 3 Department of Medical Microbiology, Medical University of Silesia, 40-752 Katowice, Poland; gmartir@sum.edu.pl; 4 Department of Transplantology, Nephrology and Internal Medicine, Medical University of Warsaw, 02-006 Warsaw, Poland; jola-md@o2.pl (J.G.); magdalena.durlik@wum.edu.pl (M.D.); 5 Department of Nephrology, Dialysis and Internal Diseases, Transplantation Outpatient Clinic, Medical University of Warsaw, 02-097 Warsaw, Poland; urszula.oldakowska@wum.edu.pl

**Keywords:** human papillomavirus, HPV, immunosuppressive therapy, immunosuppression, renal transplant recipients, liver transplant recipients, transplantation, cervical cancer, vaccination

## Abstract

Immunosuppression is a risk factor of persistent human papillomavirus (HPV) infections, which might lead to development of (pre)malignant lesions of the cervix and lower anogenital tract. Results of HPV DNA testing using cervicovaginal self-samples are comparable to those that are clinician-obtained and therefore might be used in cervical screening. The aim of this study was to assess the prevalence of high-risk HPV (hrHPV) infections, their risk factors and the genotypes distribution among women undergoing immunosuppressive therapy. Women undergoing immunosuppressive therapy for at least three months due to solid organ transplantation or autoimmune disorders were asked to self-collect samples for HPV testing using cervicovaginal brushes and complete questionnaires regarding cervical cancer risk factors. HPV DNA detection and genotyping were performed using Genotyping kit HPV GP version 2. hrHPV was detected in 26/90 (28.9%) specimens. Genotyping revealed a broad range of hrHPV, with type 16 being the most common genotype (11/26). The components of bivalent/quadrivalent or nonavalent vaccines cover all genotypes present in 4.4% and 17.8% women, respectively, and occur as a co-infection with other types in 12.2% and 23.3% of women, respectively. The only feature significantly associated with being hrHPV-positive was having at least two lifetime sexual partners. The high prevalence of hrHPV infections among immunosuppressed women emphasizes the need for regular cervical cancer screening with HPV DNA testing, which might be performed on self-collected specimen.

## 1. Introduction

Infection with high-risk human papillomavirus (hrHPV) is a risk factor of precancerous lesions and cancers of the cervix—cervical intraepithelial neoplasia (CIN) and cervical cancer—as well as intraepithelial neoplasia and cancers of the vulva, vagina and anus. Most human papillomavirus (HPV) infections are transient, resolving spontaneously within 12–24 months [1].

However, immunodeficiency is an established risk factor for persistent HPV—infections which can lead to an increased incidence of CIN in immunocompromised women. The progression of such lesions is not only more frequent but, in many cases, also more rapid. Studies comparing iatrogenically immunocompromised women due to chronic immunosuppressive therapy because of an organ transplant or autoimmune disorders (e.g., systemic lupus erythematosus, systemic sclerosis) with those due to human immunodeficiency virus (HIV) infection revealed that immunodeficiency itself is an independent risk factor for malignancy development, especially those that are virally induced [2].

HPV DNA testing is proclaimed to be a cervical cancer screening method that is superior to cytology because of its higher sensitivity and which therefore should be preferred as a primary screening method. Cervicovaginal self-sampling for HPV testing has been thoroughly investigated in recent years and is a recognized cervical cancer screening method of accuracy comparable to clinician-collected samples [3,4,5].

Among various devices tested for this purpose such as tampons, cotton swabs, cervicovaginal lavages and brushes, the latter seem to be the most reliable self-sampling devices. They are well accepted, easy to use and can be dry-stored. Therefore, if sampling is performed at home, samples can be returned in person or sent by mail in a special packaging. Moreover, self-sampling has been tested among cervical screening program non-responders and populations of underprivileged countries or residents of remote areas in order to increase the screening uptake [6,7].

The purpose of the study was to describe the prevalence of hrHPV infections, genotypes distribution and hrHPV infections’ risk factors in the group of women undergoing immunosuppressive therapy.

## 2. Materials and Methods

It was a prospective study carried out in Warsaw, Poland, aimed at women aged 18–70 years undergoing immunosuppressive therapy for at least three months, who were asked to self–collect cervicovaginal samples using the Evalyn Brush^®^ (Rovers Medical Devices, Oss, The Netherlands). The sampling kit consisted of the Evalyn Brush^®^ device, written instructions with illustrations, a plastic seal bag and an author survey concerning demographic features and cervical cancer risk factors. Sampling kits were distributed among women treated at the Department of Transplantology, Nephrology and Internal Medicine of the Medical University of Warsaw (MUW), as well as at the outpatient clinics of the 1st Department of Obstetrics and Gynecology (MUW), and the Department of General, Transplant and Liver Surgery (MUW). Exclusion criteria included menstruation on the day of enrollment (to ensure the homogeneity of the samples) or a history of anogenital tract malignancies.

Ninety-nine immunocompromised women primarily agreed to take part in the study, of which 94 returned self-collected samples. Four patients were excluded as not meeting all the inclusion criteria. Ninety samples were evaluated as eligible for final analysis. Obtained data were de-identified and participants were assigned codes.

HPV DNA detection and genotyping were performed using Genotyping kit HPV GP version 2 (Labo Bio-medical Products B.V., Rijswijk ZH, The Netherlands) enabling the qualitative identification of HPV type 6, 11, 16, 18, 26, 30, 31, 33, 35, 39, 45, 51, 52, 53, 56, 58, 59, 66, 67, 68, 70, 73 and 82.

Genotypes that were unidentifiable using the aforementioned Genotyping kit were considered as type “X”. Genotypes 6, 11, 30 and “X” were considered low risk. Genotypes 16, 18, 31, 33, 35, 39, 45, 51, 52, 56, 58 and 59 were considered high risk (hrHPV). Types 26, 53, 66, 67, 68, 70, 73 and 82 that are considered as possible/probable high-risk were defined as hrHPV in this study for analytical purposes.

To evaluate the prevalence of hrHPV in the studied population, we divided the number of hrHPV-positive women by the total number of participants (90). To evaluate the prevalence of specific hrHPV genotypes we divided the number of women in which this specific genotype was discovered (either as a single type or as a co-infection) by the total number of participants (90). Single type and multitype infections were regarded as a percentage of all hrHPV-positive women. The prevalence of genotypes included in the HPV vaccines was assessed by dividing the number of women who tested positive for the hrHPV types included in the bivalent/quadrivalent or nonavalent vaccines (without coinfection with types not included in the vaccine) by the total number of participants (90).

The hrHPV-positive women were invited for gynecological visits to the outpatient clinic of the 1st Department of Obstetrics and Gynecology (MUW). Diagnostic procedures including anogenital region and pelvic examination, as well as smear of the cervix or vaginal vault (in patients after hysterectomy), were performed at the screening visit. In case of any abnormality detection in the aforementioned procedures, women were further referred for colposcopy and biopsy when necessary. Women were then invited for regular follow-up visits and still remain under strict observation. The results of the baseline gynecological examination and colposcopy, as well as annual gynecological follow-up visits, will be presented in a forthcoming article.

The hrHPV-negative women were informed about the test result on the phone and advised to visit their gynecologists annually to undergo examination and have a Pap smear collected.

### 2.1. Statistical Analyses

Distribution of variables was assessed by the Shapiro–Wilk test. Results were presented as percentages (categorical variables), mean ± standard deviation (continuous variables). Differences between groups were calculated using the independent samples *t*-test (Student’s *t*-test) or the Mann–Whitney U-test, when appropriate. Categorical variables were compared by the chi-square test. Furthermore, in order to verify adequate statistical power, an a priori power and sample size analysis was performed. Values of *p* < 0.05 were considered as statistically significant. All analyses were performed with STATISTICA 13.1 (StatSoft, Cracow, Poland).

### 2.2. Ethical Approval

The study design was approved by the Bioethical Commission of the Medical University of Warsaw (KB/102/2015; 05.05.2015). All the participants received an information leaflet covering the details of the study and signed an informed consent.

## 3. Results

Data of 90 pharmacologically immunocompromised women were analyzed. The age range of the participants was 20–69 years, the mean age was 46.2 years. The mean duration of immunotherapy was 95.7 ± 80.8 months. The majority of women were undergoing immunosuppressive therapy in graft rejection prevention after renal transplantation (49/90, 54.4%) or liver transplantation (31/90, 34.4%), others due to conditions such as, e.g., systemic lupus erythematosus, chronic glomerulonephritis or history of heart transplantation (11/90, 12.2%). One of the women had undergone both renal and liver transplantation.

All women were receiving standard immunosuppressive therapy based on combinations of calcineurin inhibitors (tacrolimus or cyclosporine) with mycophenolate mofetil or azathioprine and corticosteroids for at least three months prior to sample collection.

hrHPV genotypes were detected in 26 out of 90 (28.9%) tested women. Type 16, existing in 11 women (12.2%), was the most common genotype in the study group. Type 18 was present in two self-samples and occurred exclusively in multitype infections. Other genotypes found in the self-samples were 31, 33, 35, 45, 56, 66, 67. The prevalence of all hrHPV types detected in the studied population is presented in Table 1.

Infection with a single hrHPV genotype was detected in 17 (65.4%) hrHPV-positive women, whereas multitype infections were found in nine hrHPV-positive women (34.6%). Type 16 was not only the most common genotype, but it was also present in the majority of multitype infections. Combinations of genotype distribution in specific multitype infections are presented in Table 2.

As vaccination against HPV is not included in the national vaccination program in Poland and is not financed by the healthcare system, only one patient from the tested group stated that she had undergone such vaccination in the past. This patient was hrHPV-negative.

The hrHPV genotypes included in bivalent/quadrivalent or nonavalent vaccines cover all genotypes present in 4/90 (4.4%) and 16/90 (17.8%) women, respectively, and were present as a co-infection with types not included in the vaccine in 11/90 (12.2%) and 21/90 (23.3%) women, respectively.

Groups of hrHPV-positive and negative women were compared in terms of features such as age, duration of immunotherapy, body mass index, habitat and education, as well as risk factors of HPV infection and cervical cancer such as age at first sexual intercourse, number of lifetime sexual partners, sexual orientation, history of sexually transmitted diseases and use of oral contraceptives. Having at least two sexual partners was significantly more common in the group of hrHPV-positive immunocompromised women. Detailed information is presented in Table 3.

There were no statistically significant percentage differences of hrHPV-positive renal transplant recipients (36.7%; 18/49) in comparison to hrHPV-positive liver transplant recipients (19.4%; 6/31; *p* = 0.984) and women using immunosuppressive drugs due to other reasons (18.2%; 2/11; *p* = 0.0238).

The most common immunosuppressive therapy regimen in the hrHPV-positive group (10/26) was tacrolimus with mycophenolate mofetil and steroids (prednisone or methylprednisolone).

Characteristics of hrHPV-positive patients are presented in Table 4.

Low-risk HPV type 6 was detected in one patient with extensive condylomata accuminata altogether with a high-risk type. In four women the genotype could not be distinguished, and these were classified as low-risk HPV type “X”.

## 4. Discussion

The prevalence of hrHPV among immunosuppressed women in the presented cohort was as high as 28.9%, while the prevalence of hrHPV in Polish women is assessed to be 14.4–25% [8,9]. Table 5 presents characteristics of studies on the prevalence of HPV among immunocompromised individuals.

A similar study to ours, investigating the genotype-specific prevalence of hrHPV in immunocompromised women based on self-sampling methods, was described by Meeuwis et al. That cohort, however, consisted exclusively of renal transplant recipients and the hrHPV prevalence was reported to be lower than in our study group (17.4%) [17]. A possible reason for this difference could be that the mean age of the participants of that study was higher than of our cohort.

A self-sampling-based study was also conducted by the same study group and described by Hinten et al. who assessed the prevalence of HPV infection in renal transplant recipients both pre- and post-transplantation, revealing an increase in prevalence of hrHPV from 19% before transplantation to 31% after transplantation [19]. This prevalence is comparable to what we discovered in our cohort.

Hinten et al. reported a statistically significant increase in hrHPV prevalence after transplantation and therefore implementation of immunosuppressive therapy, which was not related to changes in relationships or sexual behavior. Such change was not observed by the authors in the control group of women who did not undergo renal transplantation, so it is suspected that implementation of immunosuppressive therapy causes reactivation of a latent HPV infection [19].

There is, however, no agreement between authors in terms of the increase in hrHPV prevalence among immunocompromised women. Some report this prevalence to be higher than in the general population [12,13,15,16,18,19,20], whereas some find it to be comparable [9,10,14,17,21]. Morrison et al. described it to be even lower [11].

Similar to Meeuwis et al., we observed that having at least two sexual partners has a positive correlation with being hrHPV-positive [17]. Such tendency was also presented in the cohort studied by Pietrzak et al. and Roensbo et al., but the difference was insignificant [9,21]. In the study of Pietrzak, however, there was a large group of women who reported having only one sexual partner [9].

Similar to Hinten et al., we decided to consider genotypes 53, 66, 67, 68, 70 and 73, which carcinogenicity is still being discussed on by the International Agency for Research on Cancer, as hrHPV [19,22]. Immunocompromised individuals are often diagnosed with multiple genital lesions and are positive for multiple HPV types including genotypes that are generally not specific for high-grade lesions [23]. On the other hand, excluding women positive exclusively for these subtypes from final analysis (in our case subtypes 66 and 67) would result in hrHPV prevalence of 22/90 (24.4%) which remains high.

The most common genotype in our cohort was type 16, similar to Verroux and Meeuwis [15,17]. In our cohort it was detected in approximately 12.2% of women, while in the population of Polish women with normal cytology it is said to occur in 2.8% [8]. The high incidence of type 16 in our study group is particularly alarming as it is the most carcinogenic type with the highest risk of progression to CIN 3 as described by Demarco et al. [24]. This type is responsible for causing over 50% of cervical cancers [25]. Roensbo et al. described type 45 as the most prevalent, however, this constituted only two out of nine hrHPV-positive women [21].

The surprisingly low prevalence of HPV 18 (2/90) was also observed by Meeuwis et al. (2/218) and Roensbo et al. who noted no women were positive for this genotype [17,21]. However, in contrast to our cohort in the later Danish group, the prevalence of HPV 16 was also extremely low with only one case noted [21].

Not only was the hrHPV prevalence high in the studied cohort, but also a large variety of hrHPV genotypes was detected, both as single type infections as well as multitype. Multitype infections, which are considered a risk factor of HPV persistence, were detected in 34.6% of hrHPV-positive women, while Meeuwis et al. observed 27.1% of women with multitype infections [17].

As many as 23.3% of women from our cohort could benefit from being vaccinated with a nonavalent HPV vaccine. Briefly, 17.8% of women were positive exclusively for the genotypes included in the nonavalent vaccine and this group would possibly benefit from this vaccination most. However, taking into consideration the recently limited access to the nonavalent vaccine and a reported cross-protection of bivalent vaccine from non-bivalent vaccine HPV strands, vaccination with the bivalent vaccine should be advised when otherwise not possible [26]. It is worth noting that antibody titers in women immunocompromised due to solid organ transplant or autoimmune disorders might be lower when compared to those in healthy individuals and the titers might vary depending on the immunosuppressive drugs used, with lowest titers observed in patients treated with mycophenolate mofetil [27]. Unfortunately, the uptake of HPV vaccination in Poland is low, the cost high, and it is not refunded, so it is unaffordable for many citizens. Only one woman in our cohort stated a history of HPV vaccination and this person was hrHPV-negative. Vaccinating against HPV before implementation of immunosuppressive drugs should be preferred when possible. However, it seems that if a woman undergoing immunosuppressive therapy has not been vaccinated yet, it is worth performing such vaccination even in the post-implementation period. A study comparing immune response to such vaccination in women pre- and post-implementation of immunosuppressants would be extremely interesting and valuable.

The limitation of the study was the wide age range of the enrolled women (20–69 years) and that this was not a case-control study due to funding reasons. What is more, the interpretation of the acquired data in comparison to literature is limited by differences in HPV genotyping tests, age range of patients, as well as type and duration of immunosuppressive therapy.

## 5. Conclusions

A high prevalence of hrHPV and a significant proportion of multitype infections that are of higher risk of persistence and progression to precancerous lesions and cancers of the cervix and lower anogenital tract were detected in the studied population of iatrogenically immunocompromised women. This emphasizes the need for regular annual gynecological cancer screening visits and HPV DNA testing, which may be performed using self-sampling devices. Healthcare providers treating immunocompromised patients ought to educate them on cervical and lower anogenital tract cancers’ risk factors and consider advising them to limit their number of lifetime sexual partners.

As the majority of the detected hrHPV genotypes were of the nonavalent HPV vaccine—preventable types—it can be assumed that this group of women would benefit from such vaccination. Therefore, the nonavalent HPV vaccination should be recommended to immunocompromised women. As the number of women undergoing immunosuppressive therapy is increasing, implementation of a government-refunded HPV-vaccination program should be considered in such vulnerable populations, especially in the pre-transplantation period in an attempt to decrease the cervical cancer burden.

## Figures and Tables

**Table 1:** Types of detected high-risk HPV.

Genotype	16	18	31	33	35	45	56	66	67
*n*	11	2	7	3	1	6	2	3	5
%	12.2	2.2	7.8	3.3	1.1	6.7	2.2	3.3	5.6

HPV—human papillomavirus. *n*—number of women positive for a specific HPV genotype. %—percentage of women with a specific high-risk HPV genotype.

**Table 2:** Distribution of high-risk HPV genotypes.

HPV Type	*n* (%)	HPV Type	*n* (%)
16 ^1,2^	4 (4.4)	16 ^1,2^, 31 ^1^	2 (2.2)
31 ^1^	3 (3.3)	16 ^1,2^, 35	1 (1.1)
33 ^1^	3 (3.3)	16 ^1,2^, 56	1 (1.1)
45 ^1^	3 (3.3)	16 ^1,2^, 67	1 (1.1)
66	2 (2.2)	31 ^1^, 45 ^1^	1 (1.1)
67	2 (2.2)	56, 66	1 (1.1)
		16 ^1,2^, 18 ^1,2^, 45 ^1^, 67	1 (1.1)
		16 ^1,2^, 18 ^1,2^, 31 ^1^, 45 ^1^, 67	1 (1.1)

HPV—human papillomavirus; ^1^ nonavalent vaccine high-risk HPV components; ^2^ bivalent/quadrivalent vaccine high-risk HPV components.

**Table 3:** Group characteristics and risk factors of HPV infection.

	HPV+	HPV−	*p*
Age (years)	45.1 ± 13.6	46.7 ± 12.9	0.582
Duration of immunotherapy (months)	102.0 ± 68.7	93.1 ± 85.7	0.495
BMI	25.01 ± 5.31	24.18 ± 4.97	0.418
Habitat			0.156
Town > 100 000	14 (15.6%)	29 (32.2%)	
Town < 100 000	7 (7.8%)	10 (11.1%)	
Village	5 (5.6%)	25 (27.8%)	
Education			0.367
Primary	3 (3.3%)	3 (3.3%)	
Secondary	5 (5.6%)	19 (21.1%)	
Vocational	6 (6.7%)	20 (22.2%)	
Higher	12 (13.3%)	22 (24.4%)	
Age at sexual initiation (years)	20.08 ± 3.63	20.33 ± 4.56	0.763
Number of lifetime			
sexual partners (0–6)			
1	10 (11.1%)	41 (45.6%)	0.026
≥2	16 (17.8%)	23 (25.6%)	
Oral contraception users	12 (13.3%)	21 (23.3%)	0.234
Sexual orientation			0.700
Heterosexual	24 (30%)	55 (68.75%)	
Homosexual	0	0	
Bisexual	0	1 (1.5%)	
History of STDs	1 (1.2%)	1 (1.2%)	0.500
Smoking	2 (2.3%)	4 (4.5%)	0.820

HPV—human papillomavirus; HPV+—women positive for high-risk human papillomavirus; HPV−—women negative for high-risk human papillomavirus; BMI—body mass index; STDs—sexually transmitted diseases.

**Table 4:** High- risk HPV-positive patients’ characteristics.

Reason for Immunosuppressive Therapy Use	Number of High-Risk HPV-Positive Women	Duration of Immunotherapy (Months)	Immunosuppressive Therapy
Renal transplantation	18	105.4 ± 58.3	Tac, MMF, steroids = 9 (50%) Tac, steroids = 3 (16.7%) Other = 6 (33.3%)
Liver transplantation	6	63.7 ± 57.1	CsA, MMF, steroids = 3 (50%) Tac, MMF, steroids = 2 (33.3%) Tac, steroids = 1 (16.7%)
Other diseases ^1^	2	186.0 ± 144.2	MMF, Aza, steroids = 1 (50%) CsA, steroids = 1 (50%)

HPV—human papillomavirus; Tac—tacrolimus; MMF—mycophenolate mofetil; CsA—cyclosporine A; Aza—azathioprine; steroids—prednisone or methylprednisolone; ^1^ systemic lupus erythematosus and chronic glomerulonephritis.

**Table 5:** Studies on the prevalence of HPV among immunocompromised individuals.

Article	Year	Reason for Immune Compromise	Number of Women Included/Material	Mean Age (Years)	Number of Women Positive for hrHPV	Most Common hrHPV Subtype
Fairley et al. [10]	1994	RTRs	69/biopsy	Not stated	15 (22%) ^1^	Genotyping not performed
Morrison et al. [11]	1996	RTRs	21/lavage	43.9	1 (5%) ^1^	Genotyping not performed
Brown et al. [12]	2000	RTRs with lower genital tract neoplasms	20/biopsy specimens of 16 RTRs	42	11/20 (55%)	Detection of types 16 and 18 only
Seshadri et al. [13]	2001	RTRs	42/biopsy	Not stated	17 (40.5%) ^2^	PCR performed only for type 16
Paternoster et al. [14]	2008	RTRs, LTRs, R–PTRs	151/cervical smear	40	15.23% ^3^ 35% ^4^	Type 18 (*n* = 5)
Veroux et al. [15]	2009	RTRs	35/cervical smear	47 (HPV positive)	13 (37.1%) ^3^ 17 (48.6%) ^4^	Type 16 (*n* = 11, 31.4%)
Origoni et al. [16]	2011	RTRs, R–PTRs	48/cervical smear	38	10.5–27.7% (over 10 years of observation)	----
Pietrzaket al. [9]	2012	RTRs	60/cervical smear	37	11 (18.3%)	----
Meeuwiset al. [17]	2015	RTRs	218/cervicovaginal self-sample	55.4	38 (17.4%)	Type 16 (*n* = 9, 4.1%), Type 51 (*n* = 9, 4.1%) Type 66 (*n* = 7, 3.1%)
Adebamowo et al. [18]	2017	HIV+	427 (at baseline) 321 (after 6 months)/cervical smear	38	124 (29%) at baseline; 51 (15.9%) after 6 months	Type 52 (8.9% at baseline/5.5% after 6 months) Type 35 (7.0% at baseline/4.4% after 6 months)
Hinten et al. [19]	2017	RTRs	65/cervicovaginal self-sample	50 (median)	31% (SPF10-LiPA25 system)/19% (COBAS 4800)	Not stated
Cistjakovs et al. [20]	2018	RTRs	43/vaginal swabs	48 (median)	24% at 2 weeks 26% at 6 months, 36% at 1 year	Type 18 (33%)
Roensbo et al. [21]	2018	RTRs, BMTRs	60/cervical smear	55.5	15% (29.4%- BMTRs, 9.3% RTRs)	Type 45 (3.3%)

HRV—human papillomavirus, hrHPV—high risk human papillomavirus, RTRs—renal transplant recipients, PCR—polymerase chain reaction, LTRs—liver transplant recipients, R–PTRs—renal and pancreas transplant recipients, HIV+—human immunodeficiency virus positive women, BMTRs—bone marrow transplant recipients; ^1^ Genotyping not performed; ^2^ PCR performed only for HPV 16; ^3^ As stated in the article; ^4^ Types regarded as hrHPV in 2020.

## References

[1] Ho G.Y., Bierman R., Beardsley L., Chang C.J., Burk R.D. (1998). Natural history of cervicovaginal papillomavirus infection in young women. N. Engl. J. Med..

[2] Grulich A.E., Vajdic C.M. (2015). The epidemiology of cancers in human immuno-deficiency virus infection and after organ transplantation. Seminars in Oncology.

[3] Stanczuk G., Baxter G., Currie H., Lawrence J., Cuschieri K., Wilson A., Arbyn M. (2016). Clinical validation of hrHPV testing on vaginal and urine self-samples in primary cervical screening (cross-sectional results from the Papillomavirus Dumfries and Galloway—PaVDaG study). BMJ Open.

[4] Polman N.J., Ebisch R.M.F., Heideman D.A.M., Melchers W.J., Bekkers R.L., Molijn A.C., Meijer C.J., Quint W.G., Snijders P.J., Massuger L.F. (2019). Performance of human papillomavirus testing on self-collected versus clinician-collected samples for the detection of cervical intraepithelial neoplasia of grade 2 or worse: A randomised, paired screen-positive, non-inferiority trial. Lancet Oncol..

[5] Petignat P., Faltin D.L., Bruchim I., Tramèr M.R., Franco E.L., Coutlée F. (2007). Are self-collected samples comparable to physician-collected cervical specimens for human papillomavirus DNA testing? A systematic review and meta-analysis. Gynecol. Oncol..

[6] van Baars R., Bosgraaf R.P., Ter Harmsel B.W., Melchers W.J., Quint W.G., Bekkers R.L. (2012). Dry storage and transport of a cervicovaginal self-sample by use of the Evalyn Brush, providing reliable human papillomavirus detection combined with comfort for women. J. Clin. Microbiol..

[7] Yeh P.T., Kennedy C.E., de Vuyst H., Narasimhan M. (2019). Self-sampling for human papillomavirus (HPV) testing: A systematic review and meta-analysis. BMJ Glob. Health.

[8] Bardin A., Vaccarella S., Clifford G.M., Lissowska J., Rekosz M., Bobkiewicz P., Kupryjańczyk J., Krynicki R., Jonska-Gmyrek J., Danska-Bidzinska A. (2008). Human papillomavirus infection in women with and without cervical cancer in Warsaw, Poland. Eur. J. Cancer.

[9] Pietrzak B., Mazanowska N., Ekiel A.M., Durlik M., Martirosian G., Wielgos M., Kaminski P. (2012). Prevalence of high-risk human papillomavirus cervical infection in female kidney graft recipients: An observational study. Virol. J..

[10] Fairley C.K., Chen S., Tabrizi S.N., McNeil J., Becker G., Walker R., Atkins R.C., Thomson N., Allan P., Woodburn C. (1994). Prevalence of HPV DNA in cervical specimens in women with renal transplants: A comparison with dialysis-dependent patients and patients with renal impairment. Nephrol. Dial. Transplant..

[11] Morrison E.A., Dole P., Sun X.W., Wright T.C. (1996). Low prevalence of human papillomavirus infection of the cervix in renal transplant patients. Nephrol. Dial. Transplant..

[12] Brown M.R., Noffsinger A., First M.R., Penn I., Husseinadeh N. (2000). HPV subtype analysis in lower genital tract neoplasms of female renal transplant recipients. Gynecol. Oncol..

[13] Seshadri L., George S.S., Vasudevan B., Krishna S. (2001). Cervical intraepithelial neoplasia and human papilloma virus infection in renal transplant recipients. Indian J. Cancer..

[14] Paternoster D.M., Cester M., Resente C., Pascoli I., Nanhorngue K., Marchini F., Boccagni P., Cillo U., Ribaldone R., Amoruso E. (2008). Human papilloma virus infection and cervical intraepithelial neoplasia in transplanted patients. Transplantation Proceedings.

[15] Veroux M., Corona D., Scalia G., Garozzo V., Gagliano M., Giuffrida G., Costanzo C.M., Giaquinta A., Palermo I., Zappalà D. (2009). Surveillance of Human Papilloma Virus Infection and Cervical Cancer in Kidney Transplant Recipients: Preliminary Data. Transplantation Proceedings.

[16] Origoni M., Stefani C., Dell’Antonio G., Carminati G., Parma M., Candiani M. (2011). Cervical Human Papillomavirus in transplanted Italian women: A long-term prospective follow-up study. J. Clin. Virol..

[17] Meeuwis K.A., Hilbrands L.B., IntHout J., Slangen B.F., Hendriks I.M., Hinten F., Christiaans M.H., Quint W.G., Van De Kerkhof P.C., Massuger L.F. (2015). Cervicovaginal HPV infection in female renal transplant recipients: An observational, self-sampling based, cohort study. Am. J. Transplant..

[18] Adebamowo S.N., Olawande O., Famooto A., Dareng E.O., Offiong R., Adebamowo C.A. (2017). Persistent low-risk and high-risk human Papillomavirus infections of the Uterine cervix in HIV-negative and HIV-Positive Women. Front. Public Health.

[19] Hinten F., Hilbrands L.B., Meeuwis K.A., IntHout J., Quint W.G., Hoitsma A.J., Massuger L.F., Melchers W.J., de Hullu J.A. (2017). Reactivation of Latent HPV Infections after Renal Transplantation. Am. J. Transplant..

[20] Cistjakovs M., Sultanova A., Jermakova O., Sokolovska L., Chapenko S., Lesina-Korne B., Rozental R., Murovska M., Ziedina I. (2018). Importance of High-Risk Human Papillomavirus Infection Detection in Female Renal Transplant Recipients in the First Year after Transplantation. Infect. Dis. Obstet. Gynecol..

[21] Roensbo M.T., Blaakær J., Skov K., Hammer A. (2018). Cervical HPV prevalence and genotype distribution in immunosuppressed Danish women. Acta Obstet. Gynecol. Scand..

[22] IARC (2012). Working Group on the Evaluation of Carcinogenic Risks to Humans. Biological Agents. A Review of Human Carcinogens. IARC Monogr. Eval. Carcinog. Risks Hum..

[23] Hampl M., Wentzensen N., Vinokurova S., von Knebel-Doeberitz M., Poremba C., Bender H.G., Kueppers V. (2007). Comprehensive analysis of 130 multicentric intraepithelial female lower genital tract lesions by HPV typing and p16 expression profile. J. Cancer Res. Clin. Oncol..

[24] Demarco M., Hyun N., Carter-Pokras O., Raine-Bennett T.R., Cheung L., Chen X., Hammer A., Campos N., Kinney W., Gage J.C. (2020). A study of type-specific HPV natural history and implications for contemporary cervical cancer screening programs. Eclinicalmedicine.

[25] Clifford G.M., Smith J.S., Plummer M., Muñoz N., Franceschi S. (2003). Human papillomavirus types in invasive cervical cancer worldwide: A meta-analysis. Br. J. Cancer..

[26] European Centre for Disease Prevention and Control (2012). Introduction of HPV Vaccines in EU Countries—An Update.

[27] Mok C.C., Ho L.Y., Fong L.S., To C.H. (2013). Immunogenicity and safety of a quadrivalent human papillomavirus vaccine in patients with systemic lupus erythematosus: A case–control study. Ann. Rheum. Dis..

